# Revealing Genomic Profile That Underlies Tropism of Myeloma Cells Using Whole Exome Sequencing

**DOI:** 10.1155/2015/675379

**Published:** 2015-05-18

**Authors:** Youngil Koh, Daeyoon Kim, Woo-June Jung, Kwang-Sung Ahn, Sung-Soo Yoon

**Affiliations:** ^1^Department of Internal Medicine, Seoul National University Hospital, Seoul 110744, Republic of Korea; ^2^Cancer Research Institute, Seoul National University College of Medicine, Seoul 110799, Republic of Korea; ^3^Biomedical Research Institute, Seoul National University Hospital, Seoul 110744, Republic of Korea; ^4^Functional Genome Institute, PDXen Biosystem Inc., Seoul 143901, Republic of Korea

## Abstract

*Background. *Previously we established two cell lines (SNU_MM1393_BM and SNU_MM1393_SC) from different tissues (bone marrow and subcutis) of mice which were injected with single patient's myeloma sample. We tried to define genetic changes specific for each cell line using whole exome sequencing (WES).* Materials and Methods. *We extracted DNA from SNU_MM1393_BM and SNU_MM1393_SC and performed WES. For single nucleotide variants (SNV) calling, we used Varscan2. Annotation of mutation was performed using ANNOVAR.* Results.* When calling of somatic mutations was performed, 68 genes were nonsynonymously mutated only in SNU_MM1393_SC, while 136 genes were nonsynonymously mutated only in SNU_MM1393_BM.* KIAA1199, FRY, AP3B2, *and* OPTC* were representative genes specifically mutated in SNU_MM1393_SC. When comparison analysis was performed using TCGA data, mutational pattern of SNU_MM1393_SC resembled that of melanoma mostly. Pathway analysis using KEGG database showed that mutated genes specific of SNU_MM1393_BM were related to differentiation, while those of SNU_MM1393_SC were related to tumorigenesis.* Conclusion.* We found out genetic changes that underlie tropism of myeloma cells using WES. Genetic signature of cutaneous plasmacytoma shares that of melanoma implying common mechanism for skin tropism.* KIAA1199, FRY, AP3B2,* and* OPTC* are candidate genes for skin tropism of cancers.

## 1. Introduction

Multiple myeloma is a malignant B-cell disorder characterized by proliferation of atypical plasma cells in bone marrow [1] with or without the presence of monoclonal immunoglobulin protein in serum and/or urine [2]. Multiple myeloma has correlation with plasmacytoma, which is a mass of plasma cells found outside of bone marrow [3] that needs medical intervention with radiotherapy [4] or chemotherapy. While multiple myeloma frequently accompanies plasmacytoma at the time of diagnosis, plasmacytoma precedes multiple myeloma in some cases. The disease entity called solitary plasmacytoma exists in 4% of plasma cell tumors [5, 6], and approximately 40–50% of patients with solitary plasmacytoma will develop multiple myeloma [7]. Hence, plasmacytoma is an early form or an accompanying disease of myeloma, and the data regarding the “clinical behavior” of plasmacytoma are quite accumulated. However, not much is known about the cellular biology of plasmacytoma* per se*.

Fortunately, we previously succeeded in establishing two cell lines with different tropism from a single patient [8]. Briefly, 14 weeks after injecting mononuclear cells obtained from multiple myeloma patient's bone marrow in a NRG/SCID mouse via tail vein, tumor developed at subcutis of the mouse. The engraftment of myeloma cells into mouse bone marrow (BM) was also observed. After separation and culturing cells from subcutis and BM, we succeeded in the establishing of two cell lines (SNU_MM1393_SC and SNU_MM1393_BM) from a single patient with different tropism. These two cell lines showed different biologic behavior. In contrast to SNU_MM1393_BM, cell proliferation of SNU_MM1393_SC was IL-6 independent. SNU_MM1393_BM and SNU_MM1393_SC showed a high degree of resistance against bortezomib compared to U266 cell line. SNU_MM1393_BM had the greater lethality compared to SNU_MM1393_SC.

From genetic perspective, we believe that unique somatic mutations may allow tumor cells to adapt and survive in tumor microenvironment. In other words, there may be specific genetic changes that contribute to tumor tropism. Hence, in this study, we tried to characterize genomic profile specific for SNU_MM1393_BM and SNU_MM1393_SC to understand genetic background of difference in these cell lines. We were particularly interested in genetic changes specific for SNU_MM1393_SC, because we thought these SNU_MM1393_SC specific genetic changes would reveal genetic background for plasmacytoma which exhibit tropism for extramedullary space. To find these genetic changes, we performed whole exome sequencing (WES) using DNA of both cell lines. As it is well known, WES allows comprehensive characterization of genomic changes in individual tumors [9].

## 2. Materials and Methods

### 2.1. DNA Preparation and Whole Exome Sequencing

DNA was extracted from two cell lines (SNU_MM1393_BM and SNU_MM1393_SC). QuickGene DNA whole blood kit S (Kurabo Industries Ltd., Japan) was used to extract DNA according to the manufacturer's recommendations. For WES, we sequenced exome using the Solexa sequencing technology platform (HiSeq2000, Illumina, San Diego, CA, USA) following the manufacturer's instructions. We randomly sheared 3 ug of genomic DNA using Covaris System to generate about 150 bp inserts. The fragmented DNA was end-repaired using T4 DNA polymerase and Klenow polymerase, and Illumina paired-end adaptor-oligonucleotides were ligated to the sticky ends. We analyzed the ligation mixture by electrophoresis on an agarose gel, sliced and purified fragments with 200–250 bp sizes. Purified DNA library was hybridized with SureSelect Human All Exon V4 probes set (Agilent, Santa Clara, CA, USA) to capture 50Mb targeted exons following manufacturer's instruction. We prepared the HiSeq2000 paired-end flow cell to the manufacturer's protocol using captured exome library. Clusters of PCR colonies were then sequenced on the HiSeq2000 platform using recommended protocols from the manufacturer.

### 2.2. Alignment of FASTQ File

FASTQ files were aligned to human reference (human_g1k_v 37.fasta) by using the Burrows-Wheeler aligner (BWA-0.7.5) [10] to make SAM file. SortSam in Picard-tools-1.68 was used to convert to BAM file and sort with chromosome and went through a PCR duplicate marking process, which enables the Genome Analysis Toolkit (GATK-1.6.5) to ignore duplicates in subsequent processing [11]. We performed a local realignment prior to recalibration, which gives the most accurate quality scores for each sample. Recalibration was performed to increase recalibration accuracy. A default value was set for all processes.

### 2.3. Variant Calling and Annotation

For single nucleotide variant (SNV) and small indel calling, we used Varscan2 (http://genome.wustl.edu/) [12]. Because germline control was absent, we used pilup2snp command for single sample calling using human_g1k_v 37 as reference genome. We set the following options: (1) minimum coverage above value of 20, (2) minimum variant allele frequency above value of 0.1, and (3) default *p* value below 0.05 other option value set as default values. To select unique mutation, we performed comparison between two calling results. For functional annotation and prediction of variant effect, we used ANNOVAR [13] with Polyphen [14] database version 2.2.2.

### 2.4. Use of Public Database as a Reference

For comparing public data with results in this study, we used datasets from TCGA (https://tcga-data.nci.nih.gov/tcga/tcgaHome2.jsp), cBioPortal for Cancer Genomics (http://www.cbioportal.org/public-portal/), and KEGG database for pathway analysis (http://david.abcc.ncifcrf.gov/).

## 3. Results

### 3.1. Tumor Purity, Alignment, and Coverage Statistics

FastQC toolkit was used for statistical analysis. The raw data size of SNU_1393MM_BM and SNU_1393MM_SC was 9,090 MB and 8,979 MB, respectively. Approximately 99.00% of the targeted reads (165483843 reads) were covered sufficiently to pass our thresholds for calling variants (MAPQ > 20 by NGS QC Toolkitv2.3). MAPQ distribution following that above 30 was 98.2% (164088367), above 20 was 0.8% (1395476), and below 20 of MAPQ was under 10%. For SNU_1393MM_SC, MAPQ distribution following that above 30 was 98.1% (159871347), above 20 was 0.8% (154084), and below 20 of MAPQ was around 10%.

### 3.2. Somatic Mutation Calling Summary

**Figure 1:**
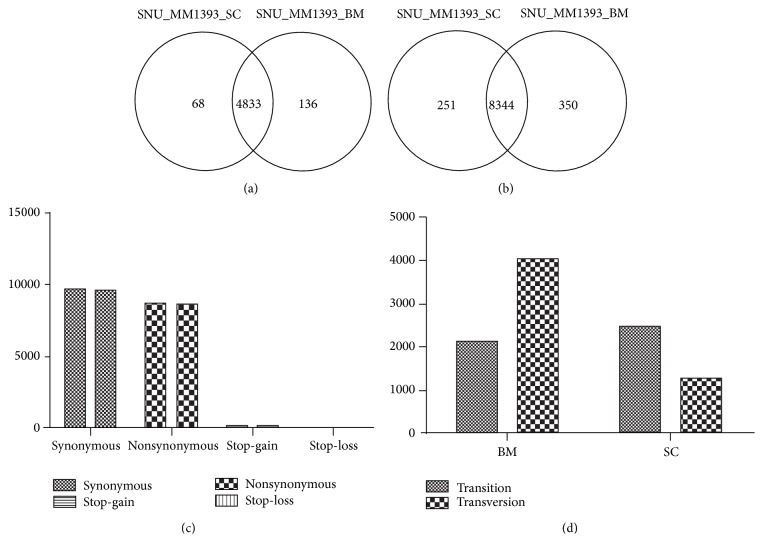


When SNV calling was performed using Varscan, a total of 18573 SNVs were found in SNU_MM1393_SC. Their distribution according to the functional consequences was as follows: 8595 (46.2%) nonsynonymous, 9575 (51.5%) synonymous, 68 (0.003%) stop-gain, and 6 (0.0003%) stop-loss. In SNU_MM1393_BM, a total of 18781 SNVs were found and their distribution was as follows: 8694 (46.2%) nonsynonymous, 9667 (51.5%) synonymous, 75 (0.004%) stop-gain, and 5 (0.0003%) stop-loss. As for nonsynonymous SNVs, we found 8595 nonsynonymous SNVs in 4901 genes for SNU_MM1393_SC, while 8694 nonsynonymous SNVs in 4969 genes were found in SNU_MM1393_BM. There was overlapping of 8344 nonsynonymous SNVs, and 251 nonsynonymous SNVs and 350 nonsynonymous SNVs were unique for SNU_MM1393_SC and SNU_MM1393_BM, respectively (Figures 1(a)–1(c)).

The rate of transversion and transition in the coding region was different between the two cell lines. While transversion was dominant event in SNU_MM1393_BM cell line, transition was dominant event in SNU_MM1393_SC. Absolute transversion rate was much higher in SNU_MM1393_BM (65.5%) than SNU_MM1393_SC (34.0%) (Figure 1(d)).

### 3.3. Comparison of Genomic Signature Using Public Database

After calling of SNVs, we compared genomic signatures of SNU_MM1393_SC and SNU_MM1393_BM with those of tumors in public database. For this comparison, we selected 12 nonsynonymous SNVs that is unique for SNU_MM1393_BM and 11 nonsynonymous SNVs that is unique for SNU_MM1393_SC. These SNVs were selected according to the criteria below: with the assumption that two cell lines consisted of single cell population, we selected genes with variant allele frequency between 0.4 and 0.6. First, the frequencies of these SNVs were investigated in open source data of multiple myeloma (Multiple Myeloma Research Consortium) [15] using cBioportal for Cancer Genomics (http://www.cBioportal.org). Around half of SNVs found in our cell lines were found with low frequency (0.5–2%) in open source database of multiple myeloma (Table 1).

**Figure 2:**
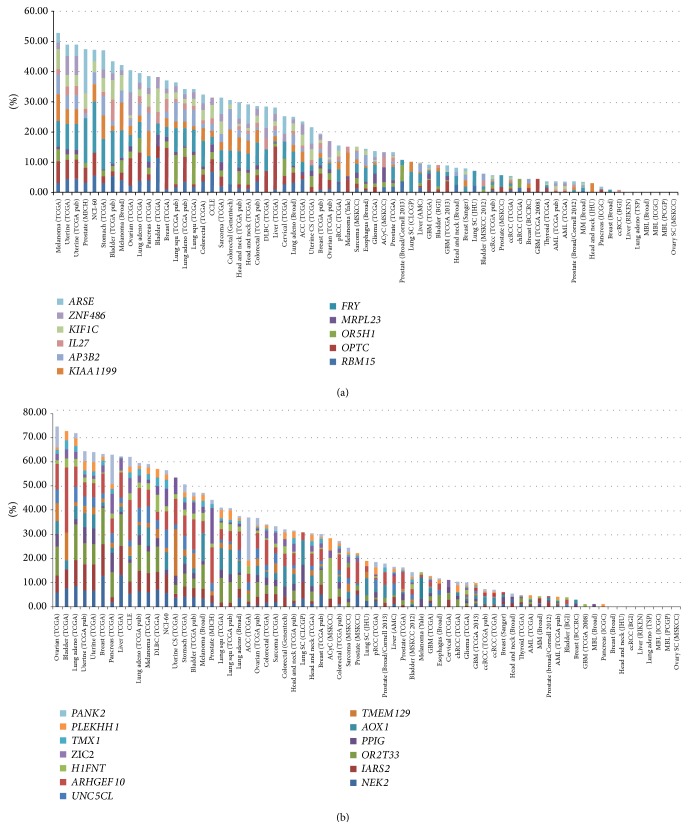


Then, the frequencies of these SNVs were investigated in open source data of various cancers (http://www.cBioportal.org). When this analysis was performed, SNVs which are unique for SNU_MM1393_SC were frequently detected in melanoma (52.8%) and uterine cancer (49.0%). On the other hand, SNVs those which are unique for SNU_MM1393_BM were frequently found in ovarian cancer (74.7%) and bladder cancer (72.8%) (Figure 2).

### 3.4. Monte Carlo Simulation and Pathway Analysis

Using Monte Carlo Simulation (Ratio of dNs/dS), we examined distribution statistics of SNVs found in two cell lines, respectively. It is believed that nonrandomness of SNV distribution is related to the functional importance of those SNVs. When this analysis was performed, ratio for SNU_MM1393_BM was 2.8 (*p* = 0.14), while it was 1.1 for SNU_MM1393_SC (*p* = 0.07). Hence, SNV distribution in both cell lines was random with cut-off *p* value of 0.05. Our results indicated that unique nonsynonymous mutations of SNU_MM1393_SC seemed biologically more neutral than those of SNU_MM1393_BM although they were statistically insignificant.

**Figure 3:**
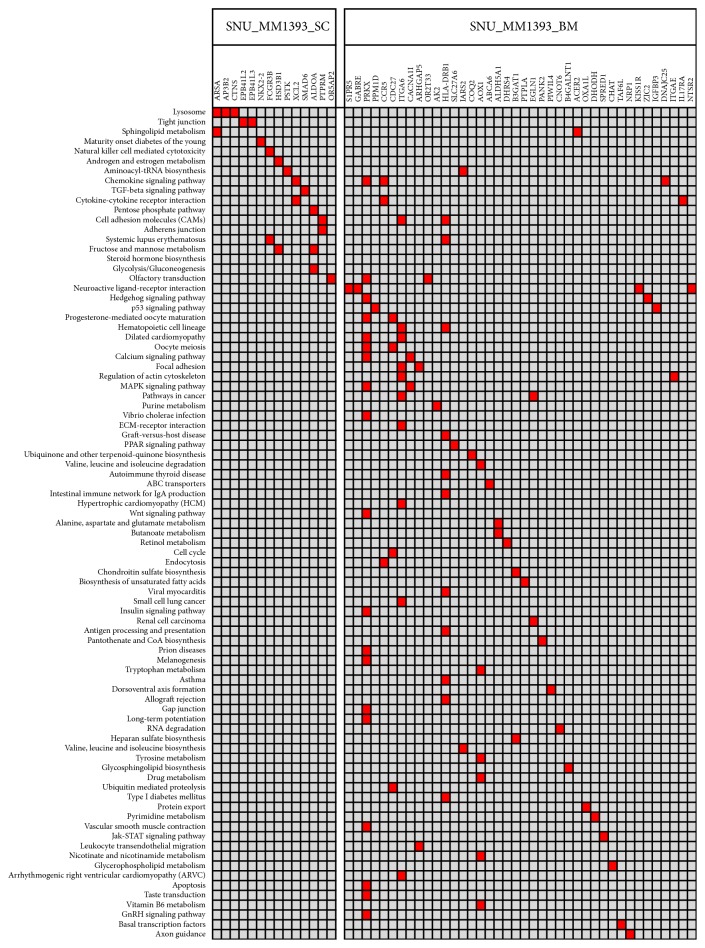


In KEGG pathway analysis of unique somatic mutation from both cell lines, the result of pathway analysis showed both similarities and differences. Chemokine signaling pathway and chemokine-chemokine interaction pathway were showed in two cell lines at the same time. While tight junction and adherens junction were shown to be involved only in SNU_MM1393_SC, SNU_MM1393_BM cell line showed diverse disorder in pathway such as cell cycle, Wnt signaling pathway, and MAPK signaling pathway (Figure 3).

## 4. Discussion

In this report, we analyzed genomic signature of two cell lines derived from single patient. These cell lines have different tropism, and SNU_MM1393_SC is plasmacytoma which has tropism for skin. Thus, we thought this study would reveal genetic changes that are related to skin tropism and plasmacytoma. In fact, recent study reported the process of segregation of genetic changes in tumor cells during clonal expansion [16]. As far as we know, this is the first WES study comparing the genomics of myeloma cells in bone marrow and plasmacytoma. Varscan2 which was used in our study for somatic variant calling is one of the most commonly used tools to detect somatic mutation along with MuTect [12, 17]. In fact, many papers already published in the field of cancer genomics already used Varscan2 [18–20].

As expected, most of SNVs (96.5%) overlapped between the cell lines, suggesting their common originality from a single patient. And the most coding sequence SNVs in both SNU_MM1393_BM and SNU_MM1393_SC were neutral with respect to adaptation and cancer cell growth. This is also supported by the outcome of Monte Carlo Simulation, where distribution of SNVs did not show significant nonrandomness. It has been suggested that bone marrow microenvironment strictly regulates the growth of cell via dynamic interplay among hematopoietic cells [21]. And our results indicate that only a small subset of nonsynonymous SNVs in cancer are affected by selection, making it possible to interpret SNV trends as reflection of underlying mutational processes.

The most interesting finding in our data is related to the genetic changes unique for SNU_MM1393_SC. We conjectured in the planning of this study that genetic change unique for SNU_MM1393_SC would be related to skin tropism and formation of plasmacytoma. And, as expected, genomic signature that is unique to SNU_MM1393_SC had more than 50% of overlapping with melanoma which is a primary skin tumor. This finding highly coincides with our conjecture and SNVs such as* KIAA1199*,* FRY*,* AP3B2*, and* OPTC* may be the very gene related to skin tropism of cancers. These 4 are the genes that are mutated in SNU_MM1393_SC and are frequently found in melanoma samples. In fact, it has been known that there are common genetics between melanoma and plasmacytoma such as CDKN2A germline mutation in prenext generation sequencing (NGS) era [22].

One more noticeable finding in our study is that the number of SNVs was higher in SNU_MM1393_BM than in SNU_MM1393_SC and transversion rate was higher in SNU_MM1393_BM than in SNU_MM1393_SC. Also dynamic gene to gene interaction in SNU_MM1393_BM was more complex than that in SNU_MM1393_SC. Along with this phenomenon and from the previous report that the number and pattern of somatic SNVs determine pathway underling cancer [23], we think that it would be biologically simpler to form a tumor in subcutis than to form a tumor in bone marrow in animal xenograft model used in our experiment. We think this is related to the fact that growth and differentiation of cancer cells in bone marrow are strictly regulated by dynamic interplay among various hematopoietic cells, compared to subcutis.

In fact, we had difficulties in the analysis of WES data due to the lack of germline reference DNA in this study. Because we used public germline database as reference in the calling of SNVs, the number of SNVs was very large compared to the previous multiple myeloma genomic studies. Moreover, it is well known that the majority of mutations observed in cancer sequencing studies are believed to be passenger mutations having little impact on the cancer cell [24]. To overcome this issue, we did not focus on the specific genetic changes found in our study. Rather, we analyzed the “pattern” of genetic changes found in our study and compared them with public database so as to find out genetic clues underlying tropism for skin and plasmacytoma. And this is also the reason why we focused on the “unique” SNVs for both SNU_MM1393_BM and SNU_MM1393_SC instead of whole genomic picture of SNU_MM1393_BM and SNU_MM1393_SC.

## Figures and Tables

**Table 1:** Gene list of two cell lines.

Gene symbol	Chromosome	Protein change	Functional effect	Variant type	Frequency in cBioportal^∗^
SNU_MM1393_BM					
*NEK2 *	1	N→S	Benign	Nonsynonymous_SNV	0%
*IARS2 *	1	None	None	Nonsynonymous_SNV	0%
*OR2T33 *	1	P→L	Possibly_damaging	Nonsynonymous_SNV	0%
*PPIG *	2	D→E	Benign	Nonsynonymous_SNV	1%
*AOX1 *	2	None	None	Nonsynonymous_SNV	1%
*TMEM129 *	4	None	None	Nonsynonymous_SNV	0%
*UNC5CL *	6	R→G	Benign	Nonsynonymous_SNV	0%
*ARHGEF10 *	8	None	None	Nonsynonymous_SNV	2%
*H1FNT *	12	R→Q	Benign	Nonsynonymous_SNV	1%
*ZIC2 *	13	None	None	Nonsynonymous_SNV	0%
*TMX1 *	14	None	None	Nonsynonymous_SNV	0%
*PANK2 *	20	G→A	Benign	Nonsynonymous_SNV	0%
SNU_MM1393_SC					
*RBM15 *	1	None	None	Nonsynonymous_SNV	0.50%
*OPTC *	1	L→P	Benign	Nonsynonymous_SNV	0%
*OR5H1 *	3	T→I	Possibly_damaging	Nonsynonymous_SNV	0%
*MRPL23 *	11	D→N	Benign	Nonsynonymous_SNV	0%
*FRY *	13	None	None	Nonsynonymous_SNV	1%
*KIAA1199 *	15	None	None	Nonsynonymous_SNV	0%
*AP3B2 *	15	None	None	Nonsynonymous_SNV	1%
*IL27 *	16	L→P	Probably_damaging	Nonsynonymous_SNV	0%
*KIF1C *	17	None	None	Nonsynonymous_SNV	1%
*ZNF486 *	19	None	None	Nonsynonymous_SNV	0%
*ARSE *	X	None	None	Nonsynonymous_SNV	1%

^∗^cBioportal data of multiple myeloma.
